# Polysaccharides extracted from *balanophora polyandra Griff* (BPP) ameliorate renal Fibrosis and EMT via inhibiting the Hedgehog pathway

**DOI:** 10.1111/jcmm.16313

**Published:** 2021-01-28

**Authors:** Luoying Li, Gang Zhou, Rui Fu, Yumin He, Li Xiao, Fan Peng, Chengfu Yuan

**Affiliations:** ^1^ College of Medical Science China Three Gorges University Yichang China; ^2^ College of Traditional Chinese Medicine China Three Gorges University Yichang China; ^3^ Yichang Hospital of Traditional Chinese Medicine Yichang China; ^4^ Department of Psychiatry and Psychology Stomatological Hospital of Jingmen City Jingmen China; ^5^ Third‐Grade Pharmacological Laboratory on Chinese Medicine Approved by State Administration of Traditional Chinese Medicine China Three Gorges University Yichang China

**Keywords:** *Balanophora polyandra Griff*, EMT, hedgehog signalling pathway, matrix metalloproteinases, renal fibrosis

## Abstract

Renal interstitial fibrosis (RIF) is a crucial pathological change leading to chronic kidney disease (CKD). Currently, no effective medicines have been available for treating it. In our research, we examined the effects of polysaccharides extracted from *Balanophora polyandra Griff* (BPPs) on kidney fibrosis and epithelial to mesenchymal transition (EMT) in vivo and in vitro, aiming to explore the underlying mechanisms. By using the mice with unilateral urethral obstruction (UUO) as experimental subjects, we examined the medicinal values of BPPs on alleviating RIF. The effects of BPPs were evaluated by examining the histological staining and relative mRNA and protein expression levels of the related genes. The possible underlying mechanisms were further explored with human normal renal proximal tubular epithelia (HK‐2 cells) as in vitro model. In UUO mice, BPP treatment could significantly alleviate interstitial fibrosis through reducing the components (Collagens I, III and IV) of extracellular matrix (ECM), and reducing the activation of fibroblasts producing these components, as revealed by inhibiting the hallmarks (fibronectin and α‐SMA) of fibroblast activation. Furthermore, BPP administration increased the expression levels of matrix metalloproteinases (MMPs) and declined those of tissue inhibitors of metalloproteinases (TIMPs). BPPs markedly ameliorated EMT in both the kidneys of UUO mice and TGF‐β1 treated HK‐2 cells. Moreover, BPP treatment decreased the expression levels of several transcriptional factors involved in regulating E‐cadherin expression, including snail, twist and ZEB1. Additionally, the Hedgehog pathway was found to be closely correlated with renal fibrosis and EMT. Altogether, our results clearly demonstrated that BPP treatment effectively inhibited the Hedgehog pathway both in renal tissues of UUO mice and TGF‐β1‐treated HK‐2 cells. Thus, BPPs ameliorated RIF and EMT in vivo and in vitro via suppressing Hedgehog signalling pathway.

## 1 INTRODUCTION

Chronic kidney disease (CKD) has become one of the important problems in global public health, as it can progress to end‐stage renal disease (ESRD), thus, affecting billions of people's health in the world.^1^ One of the important histopathologic changes in CKD is renal tubulointerstitial fibrosis characterized by deposition of large amounts of extracellular matrix (ECM), infiltration of different immune cells, disappearance of tubular epithelial cells and production of myofibroblast, leading to progressive renal impairment.^2^, ^3^, ^4^ Matrix metalloproteinases (MMPs) entail a set of proteinases, namely matrilysins, stromelysins, gelatinases, collagenases and membrane‐type MMPs,^5^ which degrade the components of glomerular basement membrane (GBM) and ECM. The activities of MMPs are regulated by a series of mechanisms, including inhibition by tissue inhibitors of metalloproteinases (TIMPs).^6^, ^7^ During the pathogenesis of fibrosis, epithelial to mesenchymal transition (EMT) occurring in epithelia of renal tubule is a part of renal fibrosis. EMT is mainly manifested by the change of cellular phenotype of epithelia, such as the decreased expression of E‐cadherin, and the acquirement of interstitial cellular phenotype, such as the elevated expression of N‐cadherin. Growing lines of evidence indicate that EMT plays a key role in the activation of renal interstitial myofibroblasts and that EMT may be the promising therapeutic targets for preventing progression of renal fibrosis.^8^, ^9^

Hedgehog signalling pathway is present in all bilaterians. In mammals, this pathway is involved in signal transduction in embryonic cells required for proper cell differentiation. Thus, this pathway is one of the key regulators for the animal development. The hedgehog protein has at least three important protein ligands, namely, Sonic hedgehog (Shh), Indian hedgehog (Ihh) and Desert hedgehog (Dhh), which are embellished by lipid. They perform functions by the way of paracrine and autocrine in their secreting areas. The Shh signalling pathway is an evolutionarily conservative pathway involved in numerous biological and pathological processes, including embryonic development, tissue repair and even tumorigenesis and development.^10^, ^11^ It has been shown that Shh signalling pathway is highly activated during the pathogenesis of fibrotic diseases, suggesting the possible existence of a potential relationship between organ fibrosis and abnormality of Shh signalling.^12^, ^13^, ^14^ Recent studies have manifested that Shh signalling pathway was also activated after renal injury and became an important mediator for progressive renal fibrosis.^12^, ^15^ Gli transcriptional factors include Gli1, Gli2 and Gli3, generally, only Gli1 can play a role in activating Shh signalling. Gli1 is a direct downstream target and receptor for Shh signalling, leading to the specific induction of renal interstitial fibroblasts (RIF). Additionally, Gli1 deficient mice could be avoided from the development of UUO‐induced RIF.^12^, ^14^ It was demonstrated that Smoothened (Smo), another important component of Shh signalling, could stimulate the activation and accumulation of Gli1. The binding of Shh ligands to a patched receptor (Ptch1) could lead to the dissociation of Smo.^16^ On contrast, when cyclopamine (CPN), an inhibitor of Smoothened receptor (Smo), was used to inhibit the inducing effect of Gli1, the RIF severity was alleviated.^12^, ^14^ Therefore, blocking the activation of Shh signalling can be helpful to inhibit RIF and prevent CKD progression.

*Balanophora polyandra Griff*, belonging to the family *Balanophoraceae*, is a natural medicinal mushroom living in association with the root system of many different host plants, especially the evergreen shrubs in Hubei, Hunan and Yunnan provinces in China.^17^ It has been demonstrated that the chemical components of *Balanophora polyandra Griff* include phenylpropanoids, flavonoids, tannins and polysaccharides etc, which have medicinal functions, such as anti‐inflammation, liver protection, anti‐oxidation and anti‐cancer as well as other biological effects.^18^ More recently, we found that *Balanophora polyandra Griff* polysaccharides (BPPs) significantly inhibited the proliferation of ovarian cancer cells via P53‐mediated pathway.^19^ However, to date, there have been no reports about the influence of BPPs on renal fibrosis and the underlying mechanisms. These deficiencies prompted us to investigate their medicinal effects on renal fibrosis in both TGF‐β1‐reduced HK‐2 cells in vitro and in kidneys of the unilateral ureteric obstruction (UUO) mice in vivo. Furthermore, we also investigated the effects of BBPs on EMT, degradation and accumulation of ECM components and the expression levels and activities of several important transcriptional factors involved in Shh signalling pathway. Our results indicated that BPPs could prevent the activation of Shh signalling pathway and thus inhibit the EMT, and finally ameliorate kidney fibrosis.

## 2 MATERIALS AND METHODS

### 2.1 Plant materials, extraction and the content of polysaccharides

The whole plant of *Balanophora polyandra Griff* was collected from Wufeng county, Hubei province, China in 2019. The sample (2W19080802) was stored in National Level‐3 Research Laboratory of Chinese Traditional Medicine Pharmacology. Preparation and determination of polysaccharide extracts were performed as previously described by Ju et al.^19^ Briefly, the dried whole plant (300 g) was chopped into small pieces and extracted with 2.0 L of distilled water by heating at 95℃ for 2.5 hours. This extraction was repeated for three times. After filtering with multiple layers of gauze, the filtrate was centrifuged at 1500 g for 10 minutes, and the supernatant fluid was collected, concentrated to 1.5 L, and precipitated with 2.5 volume of 95% ethanol (EtOH) at 4℃, overnight. Subsequently, the precipitate was dissolved in distilled water. The proteins were removed with the Sevag reagent (chloroform/butanol, 4:1). After freeze‐drying of the deproteinized solution, approximately 26.5 g of polysaccharide extract was obtained, which was stored at −20°C for subsequent experiments. The total carbohydrate content of the polysaccharide extract was determined with phenol‐sulphuric acid methods to be 54.0% (g/g) and expressed as anhydrous glucose equivalent. The detailed extraction procedures of BBPs and preliminary analysis of chemical compositions had been described in our lab's previously published papers.^19^, ^20^

### 2.2 Establishment of animal models

Fifty male specific‐pathogen‐free (SPF) C57BL/6J mice (18‐20 g) were purchased from Beijing Vital River Laboratory Animal Technology Co., Ltd (Beijing, China). The Animal Certificate was No. SCXK (Beijing) 2016‐0006. Mice were housed at the Experimental Animal Center of China Three Gorges University (Yichang, Hubei, China) at a controlled temperature of 20 ± 2℃ and humidity of 55% ± 5%. All the mice were adaptively bred for 1 week before the experiment. All the animal experiments were complied with the Experimental Animal Operating Standards of China Three Gorges University and approved by the Ethics Committee of this institute. Mice were randomly divided into five groups: sham‐operation group (Sham), unilateral ureteric obstruction group (UUO), low‐dose BPP group (UUO + BPP‐L), high‐dose BPP group (UUO + BPP‐H) and positive control group (UUO + CPN). Left ureteral ligation was performed in mice of UUO and CPN‐treated groups under sterile conditions. The mice in sham group were implemented to the uniform procedure except for ureteral obstruction. One day after the operation, the mice in the UUO + BPP‐L group and UUO + BPP‐H group were given daily by gavage with BPPs at 150 mg/kg d and 450 mg/kg d dissolved in the 0.5% solution of sodium carboxyl methylcellulose, respectively. The mice in UUO + CPN group were given with cyclopamine (Selleck Chemicals) at 5 mg/kg d by intragastric administration daily. Simultaneously, the mice in the sham group and UUO group were given separately with 0.5% solution of sodium carboxyl methylcellulose by gavage once a day. After 14 days of operation, the mice were sacrificed by asphyxiation with CO_2_, and the left kidney was dissected. A portion of the kidney was immersed in 4% paraformaldehyde for subsequent haematoxylin and eosin (H&E) staining and Masson staining. The other portion was stored at −80℃ for subsequently determining the mRNA and protein expression levels of related genes and proteins.

### 2.3 Cell culture

Human renal proximal tubular epithelial cells (HK‐2 cells) were acquired from the Cell Bank of Chinese Academy of Sciences. The Dulbecco's Modified Eagle Medium (DMEM) supplemented with 10% foetal bovine serum (FBS) (Invitrogen) was used for cell culture. These cells were pre‐treated with serum‐free DMEM containing BPPs at gradient concentrations (25, 50 and 100 μg/mL) for 24 hours, respectively. The doses were chosen according to our previous studies.^19^, ^20^ Thereafter, the cells were then induced with TGF‐β1 at 10 ng/mL for 24 hours and collected after being detached by digestion with 0.5% trypsin for 2 minutes. The un‐stimulated cells were used as the negative control group. GDC‐0449 (Vismodegib), a specific Hedgehog inhibitor, was used at 100 nM, which served as the positive control group.

### 2.4 Histopathologic evaluation

After being fixed with 4% paraformaldehyde in 0.1M PBS for 24 hours, the isolated renal tissues were conventionally embedded in paraffin. Paraffin sections of renal tissue (4 μm) were dewaxed by xylene, dehydrated by gradient ethanol (100%, 95% and 70% ethanol), and then stained with H&E. The changes in renal pathological parameters were examined under the light microscope (Olympus, BX61).

### 2.5 Masson's trichrome staining

The Masson's staining was applied in the tissue slices according to the directions of Masson's trichrome staining kit (Beyotime Institute of Biotechnology, Haimen). The bluely stained areas were quantitatively analysed by Image Pro Plus6.0 software. Twenty different visual fields were randomly selected from each section, and blue area was taken as the positive staining. The ratio of the positive area to the total field of vision was taken as the renal fibrosis index.

### 2.6 Immunohistochemistry detection

The immunohistochemical staining was employed in the paraffin‐embedded renal tissue sections. The sections from kidney (4 μm) were dewaxed by xylene, dehydrated by gradient ethanol, and co‐incubated with 0.01 M citrate buffer (pH 6.0) for antigen retrieval. The endogenous peroxidase was inactivated with 3% H_2_O_2_ and the sections were blocked using 5% bovine serum albumin (BSA) at 25℃ for 1 hour. Afterwards, slices were incubated with primary antibodies specific to α‐SMA (Santa Cruz Biotechnology, sc‐53142, 1:500) and fibronectin (Santa Cruz Biotechnology, sc‐29011, 1:500) at 4℃ overnight. The slices were washed with a PBS buffer, and incubated with corresponding secondary antibodies at 25°C for 1h. The slices were stained with freshly prepared diaminobenzidine (DAB) solution. After being counterstained with haematoxylin, the sections were differentiated with hydrochloric acid alcohol, dehydrated with gradient ethanol, and transparentized with xylene. Finally, the positive expressions of α‐SMA and fibronectin were observed under the microscope. Ten different fields were randomly picked from each slice, and the relative positively stained area was acquired using Image Pro Plus 6.0 software.

### 2.7 Quantitative real‐time PCR

The total RNA was extracted from mouse renal tissue and HK‐2 cells with Trizol reagent (Invitrogen) by following the instructions. The concentration of total RNA was assayed with Nanodrop at 260 nm and its purity was evaluated by the ratio of OD_260nm_/OD_280_ nm. The RNA integrity was detected by running electrophoresis of RNA samples on 1.2% agarose gel after treatment with DNase. 2 μg total RNA was used for the reverse transcription to synthesize template cDNA. Real‐time PCR assay was performed as follows: initial reaction at 95℃ for 10 minutes, then followed by 40 cycles of 95℃ for 15 seconds and 60℃ for 1 minutes. The 2^‐ΔΔCt^ formula was used for the analysis of expression levels of related genes, and GAPDH was taken as the internal control. The sequences of specific primers for RT‐PCR were listed in the supplementary Table S1.

### 2.8 Western blot analysis

The renal tissues or HK‐2 cells were homogenized with radioimmunoprecipitation assay (RIPA) buffer containing phenylmethylsulfonyl fluoride (PMSF, 100 mmol/L) and inhibitor of protein phosphatases. The homogenate was centrifugated at 12 000 *g* and 4℃ for 15 minutes, and then the supernatant was saved and stored at −80℃. Protein content was assayed with Bicinchoninic Acid (BCA) protein kit. A total of 50 g protein sample was diluted with 5 x loading buffer and denatured by heating at 100℃ for 5 minutes. The denatured protein samples were loaded and electrophoresed via sodium dodecyl sulphate‐polyacrylamide gel electrophoresis (SDS‐PAGE), then transferred to polyvinylidene difluoride (PVDF) membrane, which were soaked in 5% skim milk for 1h, then incubated with corresponding primary antibody at 4℃ overnight. The membranes were washed with Tris‐buffered saline, 0.1% Tween® 20 detergent (TBST) buffer for 3 times (10 minutes for each), and then incubated with the corresponding horseradish peroxidase‐labelled secondary antibody at room temperature for 1h. Lastly, after being washed, the membrane was developed with enhanced chemiluminescence (ECL) reagent and then protein bands were visualized. The expression level of β‐actin was employed for the internal normalization of each proteins. The relative expression level was expressed as the folds of induction or folds of suppression in relative to that of the corresponding control group.

### 2.9 Statistical analysis

All the experimental data were expressed with mean ± standard deviation (SD). The experimental data were processed and analysed using softwares, including Image‐proplus 6.0, GraphPad Prism 7 and SPSS13.0 software,respectively. The significance of the difference in the average values between groups was analysed by one‐way ANOVA and t‐test, and the difference between groups with *P* < .05 was considered statistically significant.

## 3 RESULTS

### 3.1 Effects of BPPs on kidney morphology in UUO mice

**FIGURE 1:**
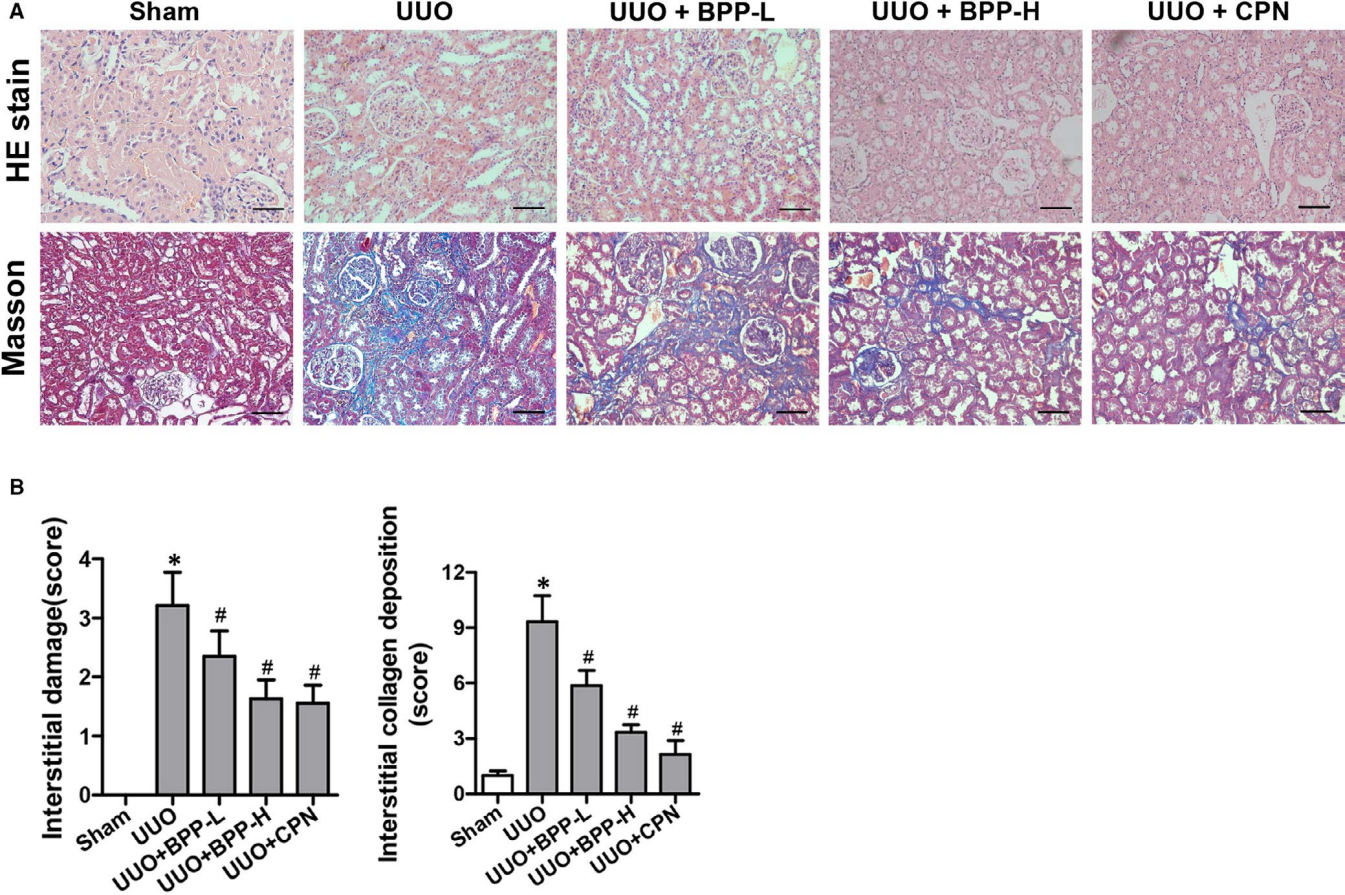
Effects of BPP on renal morphology in unilateral ureteral obstructed(UUO) mice. (A) Representative images of H&E and masson's trichrome stain of renal tissue (200 ×). (B) The statistical analyses of tubulointerstitial damage scores and average percentage of positive Masson's trichrome stained cells (blue). Data in (B) are expressed as mean ± SD, *P < .05 compared with the sham group, ^#^ *P* < .05 compared with the UUO group, n = 10. BPP‐L, low dose of BPP (150mg/kg.d); BPP‐H, high dose of BPP(450mg/kg.d); CPN, cyclopamine

In comparison with the sham group, the H&E staining of renal tissues of mice in the UUO group revealed obvious tubular dilation, atrophy and inflammatory infiltration in the renal interstitial area. Meanwhile, the Masson staining results also showed more obvious collagen deposition in the obstructed kidneys (Figure 1A and B). With BPP administration, the renal tubular injury was improved as reflected by the gradual reduction of interstitial damage scores in both UUO + BPP‐L and UUO + BPP‐H group. The collagen deposition was also remarkably declined dose‐dependently. The improved renal tubular injury and the declined collagen deposition in UUO‐BPP‐H group were closer than UUO‐BPP‐L group to that of the positive control (UUO + CPN) group (Figure 1A and B).

### 3.2 Effects of BPP on the expression levels of collagen I, collagen III, collagen IV, fibronectin and α‐SMA in the renal tissues of UUO mice

**FIGURE 2:**
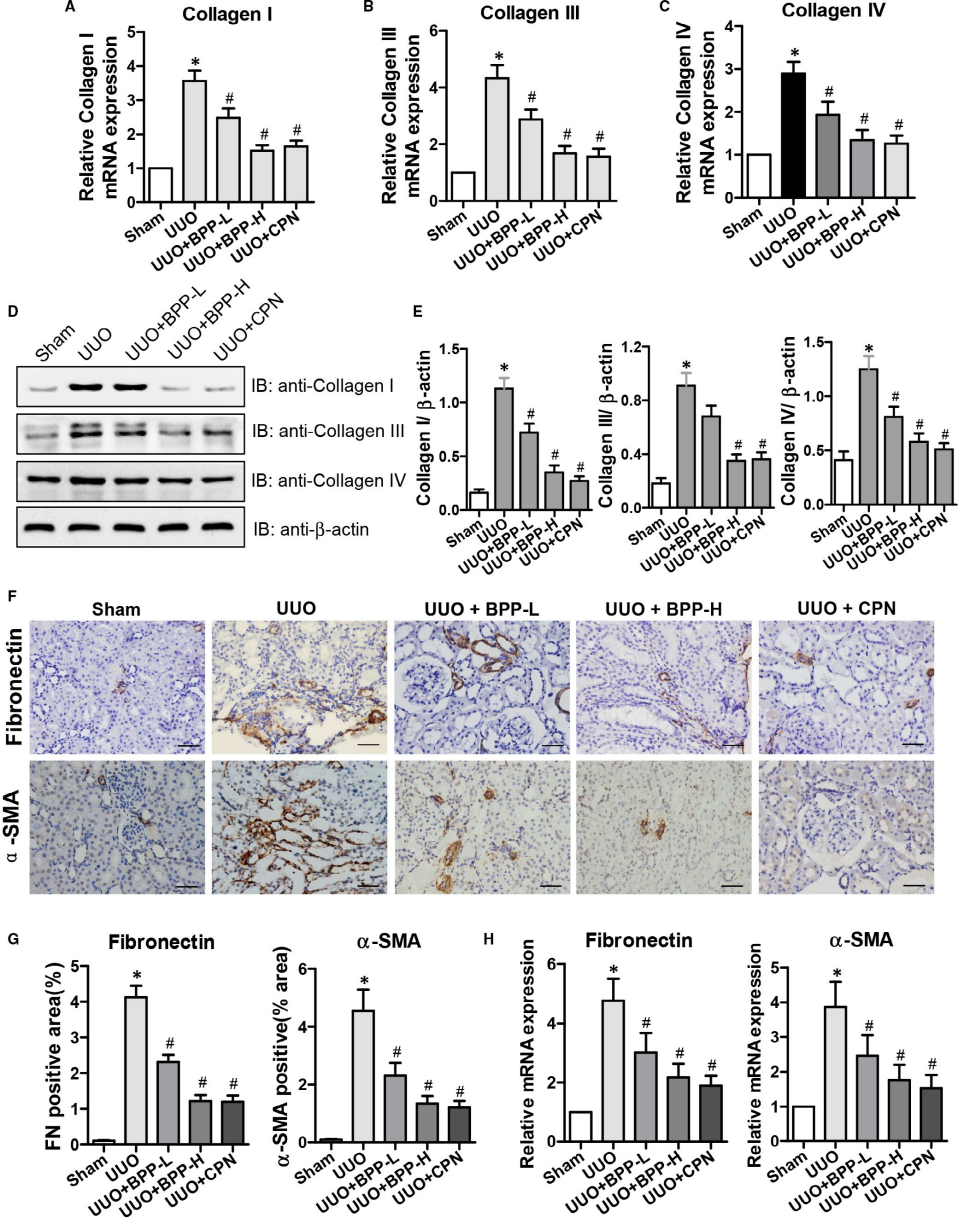
Effects of BPP on the expression levels of Collagen I, Collagen III, Collagen IV, Fibronectin and α‐SMA in the renal tissue of unilateral ureteral obstructed(UUO) mice. The mRNA expression level of Collagen I(A), Collagen III (B) and Collagen IV(C) were shown. (D) and (E) Representative results of western blot and quantitative analysis with antibodies against Collagen I, Collagen III and Collagen IV. β‐actin was used as the internal control for normalization of protein loading. (F) Fibronectin and α‐SMA protein expression levels in the renal tissue of UUO mice were detected by immunohistochemistry (400 ×). (G) The quantitative analysis for protein expression levels (positive areas) of fibronectin and α‐SMA in the kidneys. (H) The relative mRNA expression levels (folds) of Fibronectin and α‐SMA in the kidneys. All the experiments were performed in triplicate. Data in (A‐C), (E),(G) and (H) are expressed as mean ± SD, *P < .05 compared with the sham group, ^#^ *P* < .05 compared with the UUO group, n = 10. BPP‐L, low dose of BPP (150mg/kg.d); BPP‐H, high dose of BPP(450mg/kg.d); CPN, cyclopamine

Above Masson's staining results clearly showed that BPP could significantly reduce collagen deposition,and thus, inhibit the renal fibrosis in UUO mice. ECM deposition is regarded as the dominating characteristics of kidney fibrosis. Collagens I, III and IV are the major components of ECM. Therefore, we next determined the effects of BPP on the mRNA and protein levels of these collagen molecules in the renal tissues of UUO mice. The mRNA expression levels of Collagen I (Figure 2A), Collagen III (Figure 2B), Collagen IV (Figure 2C) and their corresponding protein levels (Figure 2D‐E) in the kidneys of UUO group were all dramatically and significantly increased as compared to those in the sham group (*P* < .05). In comparison with those in the UUO group, both mRNA and protein expression levels of the corresponding genes were all observably and significantly reduced dose‐dependently in the kidneys of mice in UUO + BPP‐L and UUO + BPP‐H groups with their levels in the high‐dose (BPP‐H) group being closer or similar to those in the positive control cyclopamine (CPN) group. These results demonstrated that BPP treatment could effectively decrease the expression levels of collagens I, III and IV in the renal tissues of UUO mice.

Fibroblasts are the main cells that produce ECM during renal fibrosis. Collagens I, III and IV are mostly derived from fibroblasts in the renal stroma. α‐SMA is one of the hallmarks for the activation of fibroblasts, and fibronectin is one of the most important components in the accumulation of ECM. Therefore, we subsequently examined the effects of BBPs on the protein and mRNA levels of fibronectin and α‐SMA in kidney tissues by immunochemical staining and quantitative RT‐PCR, respectively. The results of immunochemical staining of fibronectin and α‐SMA and their mRNA levels were shown in Figure 2 (F, G and H), respectively, which displayed that both the protein and mRNA levels of fibronectin and α‐SMA in the UUO group were significantly increased as compared to those in the sham group (*P* < .05). However, their mRNA and protein levels were significantly decreased after BPP treatment (*P* < .05), especially, their levels in the high‐dose (BPP‐H) group were closer than those in BPP‐L group to those in the positive control CPN group.

### 3.3 Effects of BPP on the expression Levels of MMP2, MMP9, TIMP1 and TIMP2 in the renal tissues of UUO mice

**FIGURE 3:**
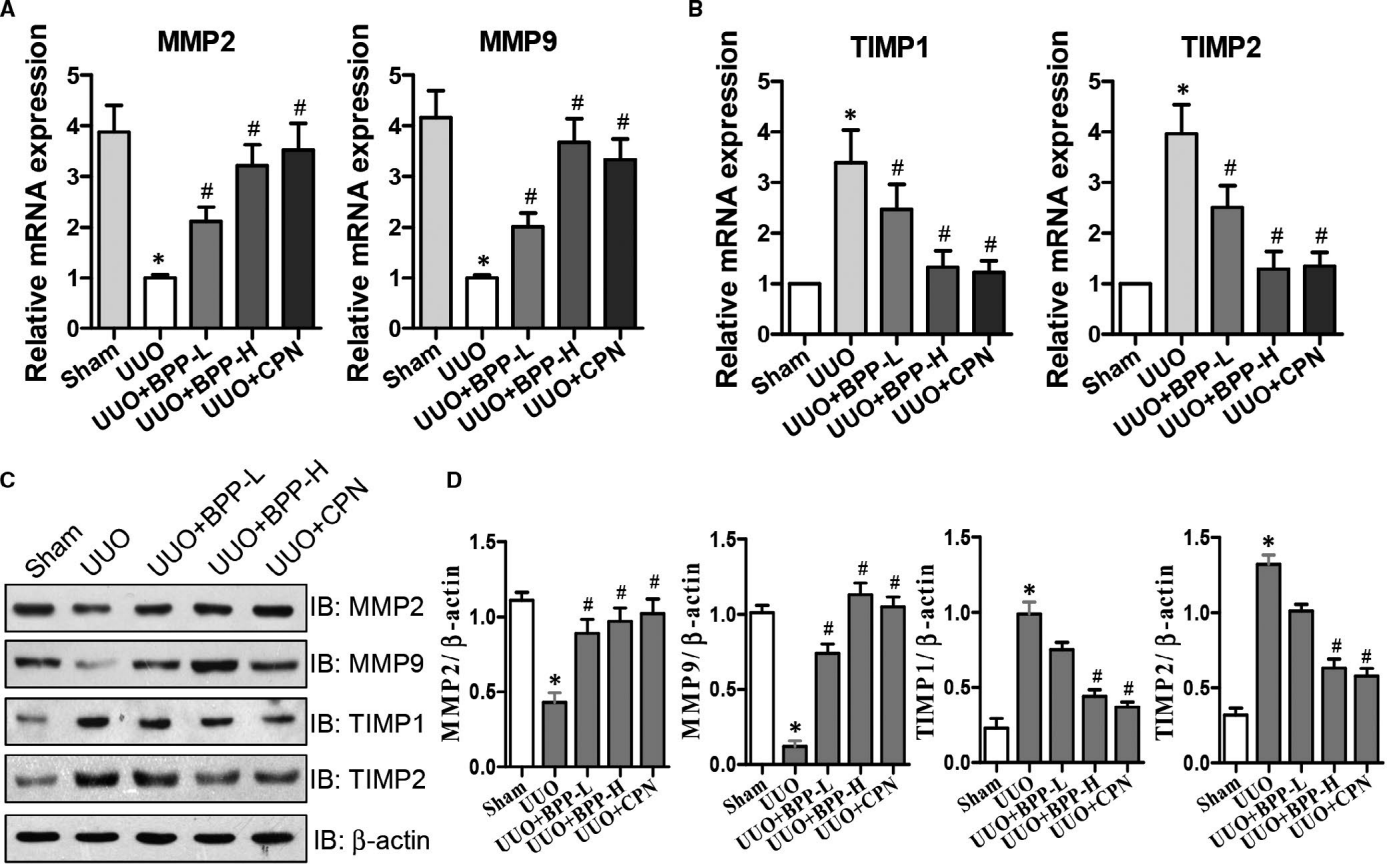
Effects of BPP on the expression levels of MMP2, MMP9, TIMP1 and TIMP2 in the renal tissue of UUO mice. (A and B) The mRNA expression levels were analysed by quantitative real‐time PCR for MMP2, MMP9, TIMP1 and TIMP2. (C) Effects of BPP on the protein expression levels of MMP2, MMP9, TIMP1 and TIMP2 as examined with their specific antibodies via western blot. Antibody against β‐actin was used as an internal control. (D) The quantitative analysis for the relative protein expression levels of MMP2, MMP9, TIMP1 and TIMP2 based on western blot results. All the experiments were performed in triplicate. Data in (A‐B) and (D) were expressed as mean ± SD, *P < .05 compared with the sham group, ^#^ *P* < .05 compared with the UUO group, n = 10. BPP‐L, low dose of BPP (150mg/kg.d); BPP‐H, high dose of BPP(450mg/kg.d); CPN, cyclopamine

The ECM degradation is mainly catalysed by MMPs, including MMP2 and MMP9, which are related to renal fibrosis. On the other hand, the activation of MMPs is regulated by TIMPs, such as TIMP1 and TIMP2. Thus, the balance between accumulation and disposition of ECM is regulated by the relative activities of MMPs and TIMPs. Above results have already proven that BPP could inhibit ECM deposition. Therefore, we next determined the effects of BPP on the levels of MMP2, MMP9, TIMP1 and TIMP2 in the kidneys of UUO mice. The results of the effects of BPP on mRNA and protein levels of MMP2 and MMP9, and those of TIMP1 and TIMP2 were shown in Figure 3(A, B, C and D), respectively. The mRNA (Figure 3A) and protein (Figure 3C and 3D) expression levels of MMP2 and MMP9 were markedly and significantly reduced in the renal tissues of UUO group in comparison with those in the sham group, while their expression levels were significantly elevated after BPP treatment (*P* < .05), especially, their levels in the high‐dose (BPP‐H) group were closer or similar to those in the positive control CPN group. However, the mRNA (Figure 3B) and protein (Figure 3C and 3D) levels of TIMP1 and TIMP2 were significantly increased in UUO group in comparison with those in the sham group, while their mRNA and protein levels were remarkably and significantly decreased after BPP treatments with those in BPP‐H group being lower and more similar to those of the positive control CPN group.

### 3.4 Effects of BPP on the EMT in the renal tissues of UUO mice

**FIGURE 4:**
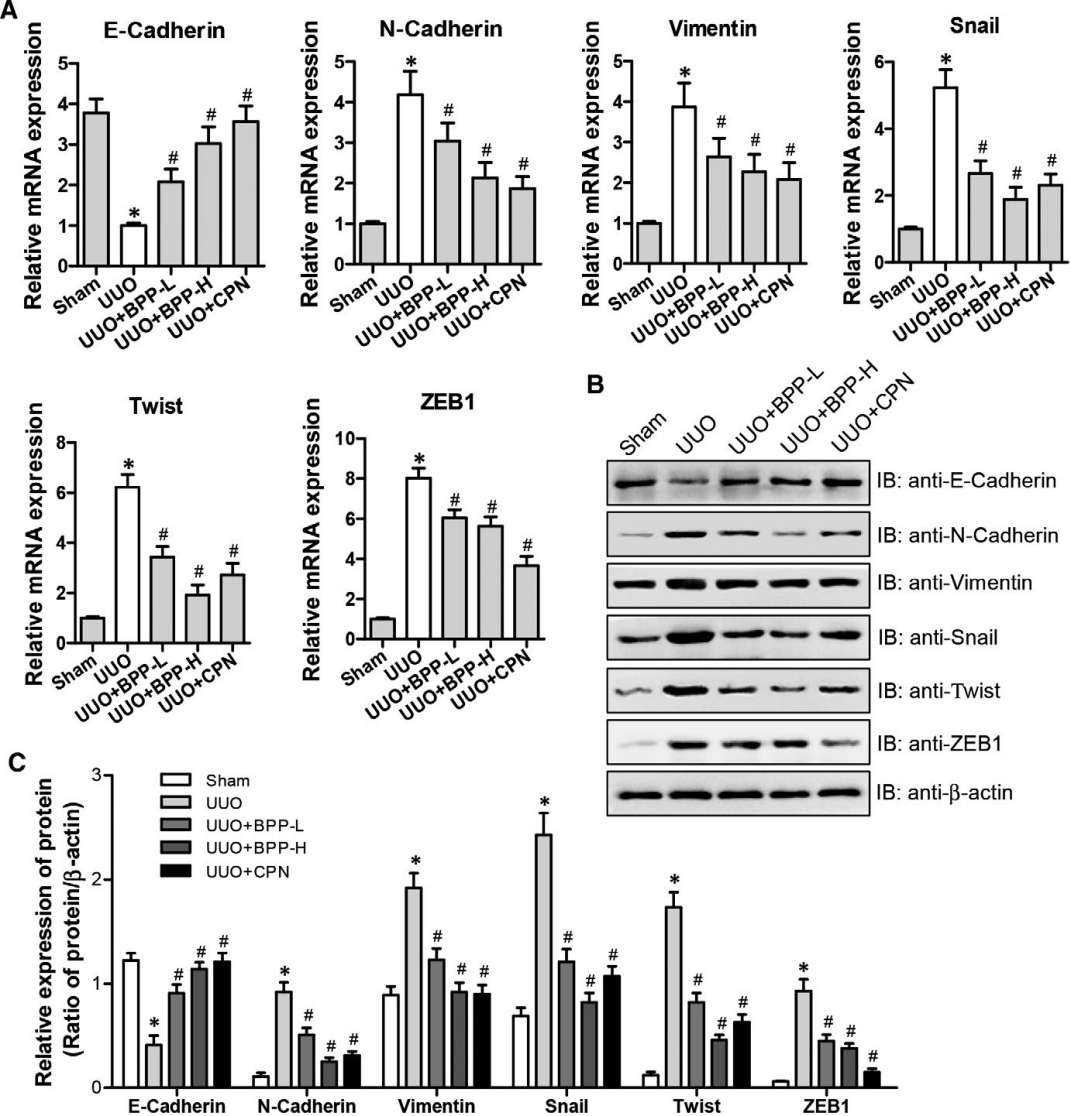
Effects of BPP on EMT in the renal tissue of UUO mice. (A) The mRNA expression levels were analysed by quantitative real‐time PCR for E‐Cadherin, N‐Cadherin, Snail, Vimentin, Twist and ZEB1 in different group. (B) The representative protein expression levels of E‐Cadherin, N‐Cadherin, Snail, Vimentin, Twist and ZEB1 were determined by western blot. Antibody against β‐actin was used as an internal control. (C) The relative protein expression levels of E‐Cadherin, N‐Cadherin, Snail, Vimentin, Twist and ZEB1 were analysed based on western blot results. All the experiments were performed in triplicate. Data in (A) and (C) were expressed as mean ± SD, *P < .05 compared with the sham group, ^#^ *P* < .05 compared with the UUO group, n = 10. BPP‐L, low dose of BPP(150mg/kg.d); BPP‐H, high dose of BPP(450mg/kg.d); CPN, cyclopamine

It has been shown that the EMT has a potential effect on RIF development.^8^, ^9^ E‐cadherin is an epithelial marker while both N‐cadherin and vimentin are the mesenchymal markers. Thus, we examined the effects of BPPs on the mRNA and protein levels of E‐cadherin, N‐cadherin and vimentin in renal tissues of UUO mice. The results were presented in Figure 4, which demonstrated that the mRNA and protein levels of E‐Cadherin were significantly lower in UUO mice than in Sham mice (Figure 4A‐C). Both BPP treatments markedly elevated the mRNA (Figure 4A) and protein levels (Figure 4B and 4C) of E‐cadherin in the kidney tissue of UUO mice, and decreased the mRNA (Figure 4A) and protein levels (Figure 4B and 4C) of N‐cadherin and vimentin as compared to those in UUO mice with those in BPP‐H group being lower, which is closer or more similar to those of the positive control CPN group.

Snail, ZEB1 and twist are the important transcriptional factors involved in regulating the expression of E‐Cadherin. Therefore, we next examined the mRNA and protein levels of snail, ZEB1 and twist. The mRNA levels of snail, ZEB1 and twist (Fig, 4A) and their protein levels (Figure 4B and 4C) were significantly higher in UUC mice than in Sham mice. However, BPP treatments significantly and dose‐dependently reduced their mRNA and protein levels in renal tissue of UUO mice with their levels in BPP‐H group being closer or more similar to those of the positive control CPN group. Above results (Figure 4 A‐C) showed that BPP treatment was capable of inhibiting the levels of snail, ZEB1 and twist in the kidney tissue of UUO mice, indicating that BPPs can alleviate EMT in the kidneys of UUO mice, and that these effects may be correlated with the down‐regulation of transcriptional factors, including snail, ZEB1 and twist.

### 3.5 Effects of BPPs on the expression levels of Shh, Gli1 and Smo involved in SHH signalling pathway in the renal tissues of UUO mice

**FIGURE 5:**
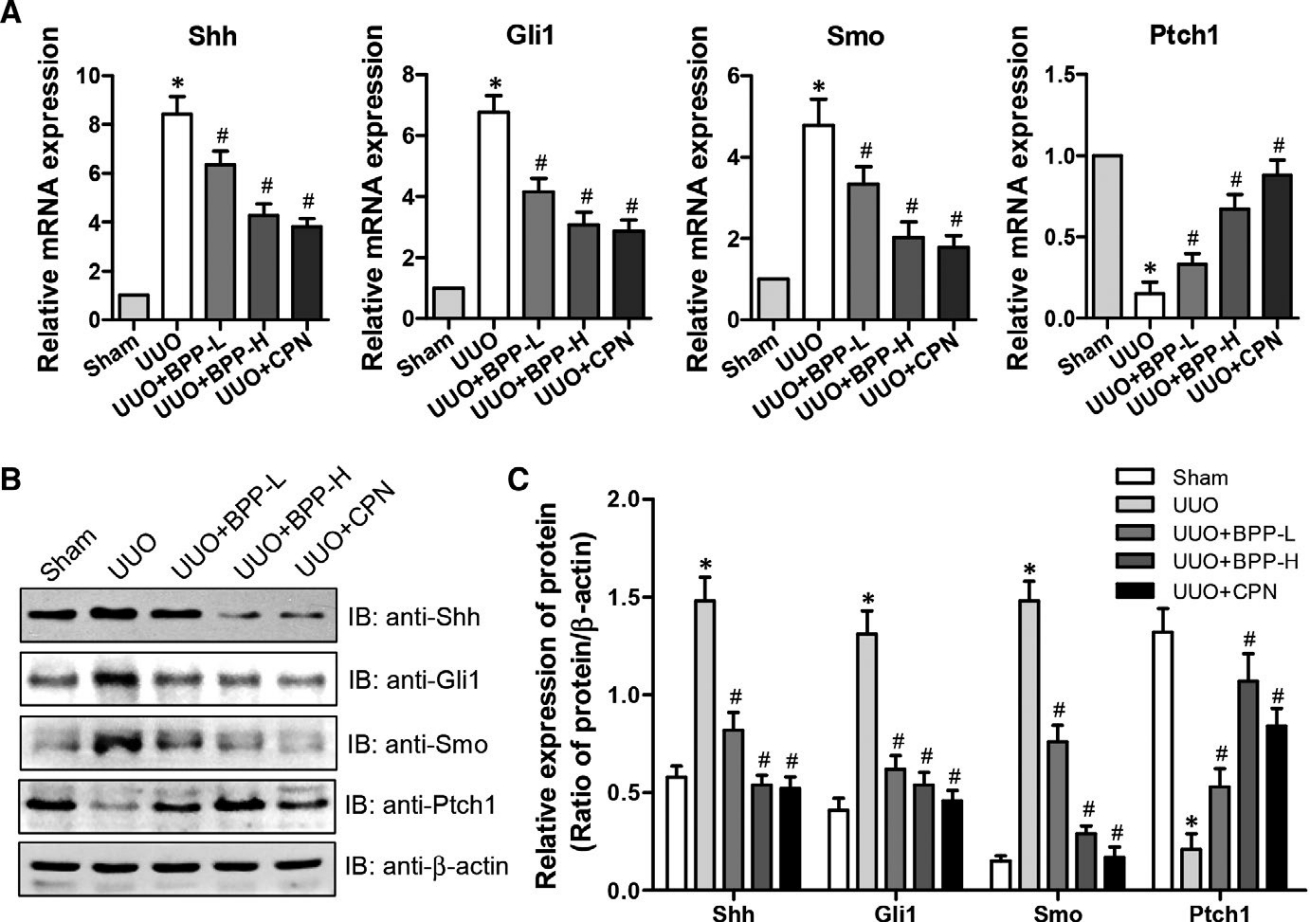
Effects of BPP on the SHH signalling pathway in the renal tissues of UUO mice. (A) The mRNA expression levels of Shh, Gli1, Ptch1 and Smo were determined by quantitative real‐time PCR. (B) The representative protein expression levels of Shh, Gli1, Ptch1 and Smo were examined by western blot. Antibody against β‐actin was used as an internal control. (C) The relative protein expression levels of Shh, Gli1, Ptch1 and Smo were analysed based on western blot results. All the experiments were performed in triplicate. Data in (A) and (C) were expressed as mean ± SD, *P < .05 compared with the sham group, ^#^ *P* < .05 compared with the UUO group, n = 10. BPP‐L, low dose of BPP(150mg/kg.d); BPP‐H, high dose of BPP(450mg/kg.d); CPN, cyclopamine

Recent studies have demonstrated that the SHH signalling pathway is activated after renal injury and is a crucial mediator of the progression of fibrosis.^12^, ^14^, ^15^ Shh, Gli1 and Smo are the important factors involved in the SHH signalling pathway while Ptch1 is an inhibitor of hedgehog. Thus, we determined the effects of BPPs on their expression levels in kidneys of the UUO mice and the results were presented in Figure 5, which showed that the mRNA (Figure 5A) and protein (Figure 5B and 5C) levels of Shh, Gli1 and Smo were significantly elevated in UUO mice as compared to those in Sham mice. Both BPP treatments dose‐dependently and significantly reduced their mRNA (Figure 5A) and protein (Figure 5B and 5C) levels in renal tissue of UUO mice. The inhibitory effects of high‐dose of BPPs were stronger than those of the low‐dose of BPP and closer to those of positive control CPN group. However, the decreased mRNA and protein levels of Ptch1 were observed in UUO mice. Both BPP treatments dose‐dependently and significantly reduced its mRNA (Figure 5A) and protein (Figure 5B and 5C) levels in renal tissue of UUO mice. The stimulating effects of high‐dose BPP were more potent than those of the low‐dose BPP and closer to those of positive control CPN group. Based on these results, we speculated that SHH signalling pathway could be activated in the kidneys of UUO mice, and that the anti‐fibrotic effects of BPP treatment might be related with the inhibition of SHH signalling pathway activated by UUO.

### 3.6 Effects of BPP on ECM accumulation and EMT in TGF‐β1‐treated HK‐2 cells

**FIGURE 6:**
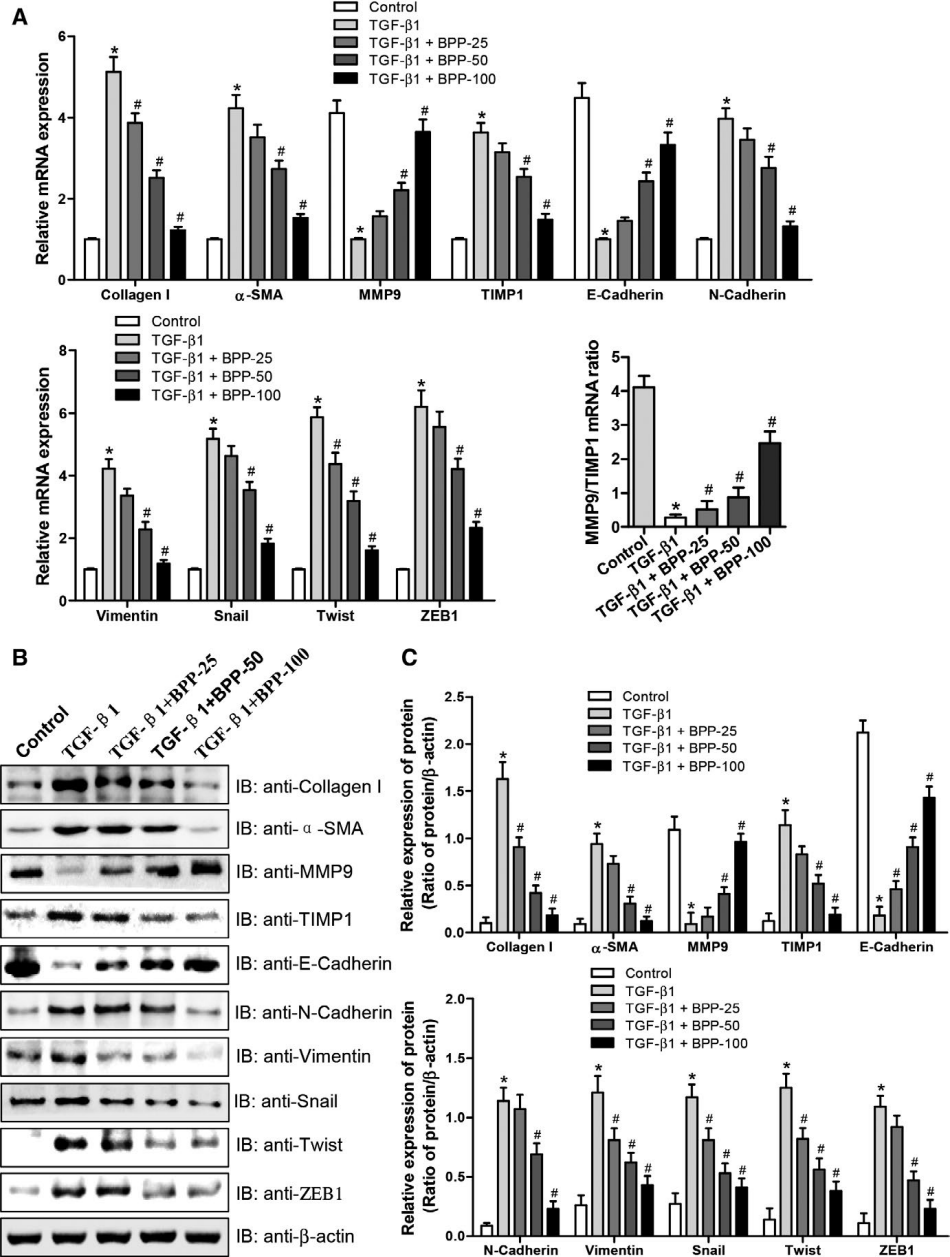
Effects of BPP on ECM accumulation and EMT in TGF‐β1‐treated HK‐2 cells. (A) The mRNA expression levels of Collagen I, α‐SMA, MMP9, TIMP1, E‐Cadherin, N‐Cadherin, Vimentin, Snail, Twist and ZEB1 were determined by quantitative real‐time PCR. (B) The representative western blot results were shown the protein expression for Collagen I, α‐SMA, MMP9, TIMP1, E‐Cadherin, N‐Cadherin, Vimentin, Snail, Twist and ZEB1. Antibody against β‐actin was used as an internal control. (C) The relative protein expression levels of Collagen I, α‐SMA, MMP9, TIMP1, E‐Cadherin, N‐Cadherin, Vimentin, Snail, Twist and ZEB1 were analysed based on western blot results. All the experiments were performed in triplicate. Data in (A) and (C) were expressed as mean ± SD, *P < .05 compared with the control group, ^#^ *P* < .05 compared with the TGF‐β1‐treated group, n = 3. BPP‐25, 25 μg/mL of BPP; BPP‐50, 50 μg/mL of BPP; BPP‐100, 100 μg/mL of BPP

It has been documented that TGF‐β1 may be the trigger of the ECM accumulation and inducer of EMT in the fibrotic kidney.^21^ For further confirming the effects in vivo of BPP on renal fibrosis described above, we next determined the changes of ECM accumulation and EMT induction by examining the effects of BPPs on the mRNA and protein levels of Collagen I, α‐SMA, MMP9, TIMP1, E‐cadherin, N‐cadherin, vimentin, snail, twist and ZEB1 in HK‐2 cells treated with both BPP and TGF‐β1. The results were shown in Figure 6. It could be seen from Figure 6 that (a) TGF‐β1 could elevate the mRNA (Figure 6A) and protein (Figure 6B and 6C) levels of collagen I, α‐SMA, TIMP1, N‐cadherin, vimentin, snail, twist and ZEB1; and (b) could suppress mRNA (Figure 6A) and protein (Figure 6B) levels of MMP9 and E‐cadherin in HK‐2 cells. Co‐treatment with BPPs dose‐dependently and significantly reversed these stimulatory or suppressive effects on the mRNA (Figure 6A) and protein levels (Figure 6B and 6C) of the corresponding genes (α‐SMA, vimentin and N‐cadherin) in HK‐2 cells. However, the mRNA and protein levels of E‐cadherin were declined, respectively. The mRNA and protein levels of Snail, Twist and ZEB1 were also observably and significantly augmented in TGF‐β1‐treated HK‐2 cells. However, the ECM accumulation and EMT transition in TGF‐β1‐treated HK‐2 cells were reversed by BPP treatment dose‐dependently. Meanwhile, the destroyed balance between MMP9 and TIMP1 was improved following BPP treatment (Figure 6 A‐C). The stimulating and inhibitory effects of BPP on the mRNA (Figure 6A) and protein expression levels of the corresponding genes in TGF‐β1‐treated HK‐2 cells were quite similar to those of the renal tissue in UUO mice. All the above results together indicated that BPP could inhibit the EMT induction and ECM accumulation in HK‐2 cells, which was accordant with the results in vivo.

### 3.7 Effects of BPP on the expression levels of Shh, Gli1, Smo and Ptch1 involved in SHH signalling pathway in TGF‐β1‐treated HK‐2 cells

**FIGURE 7:**
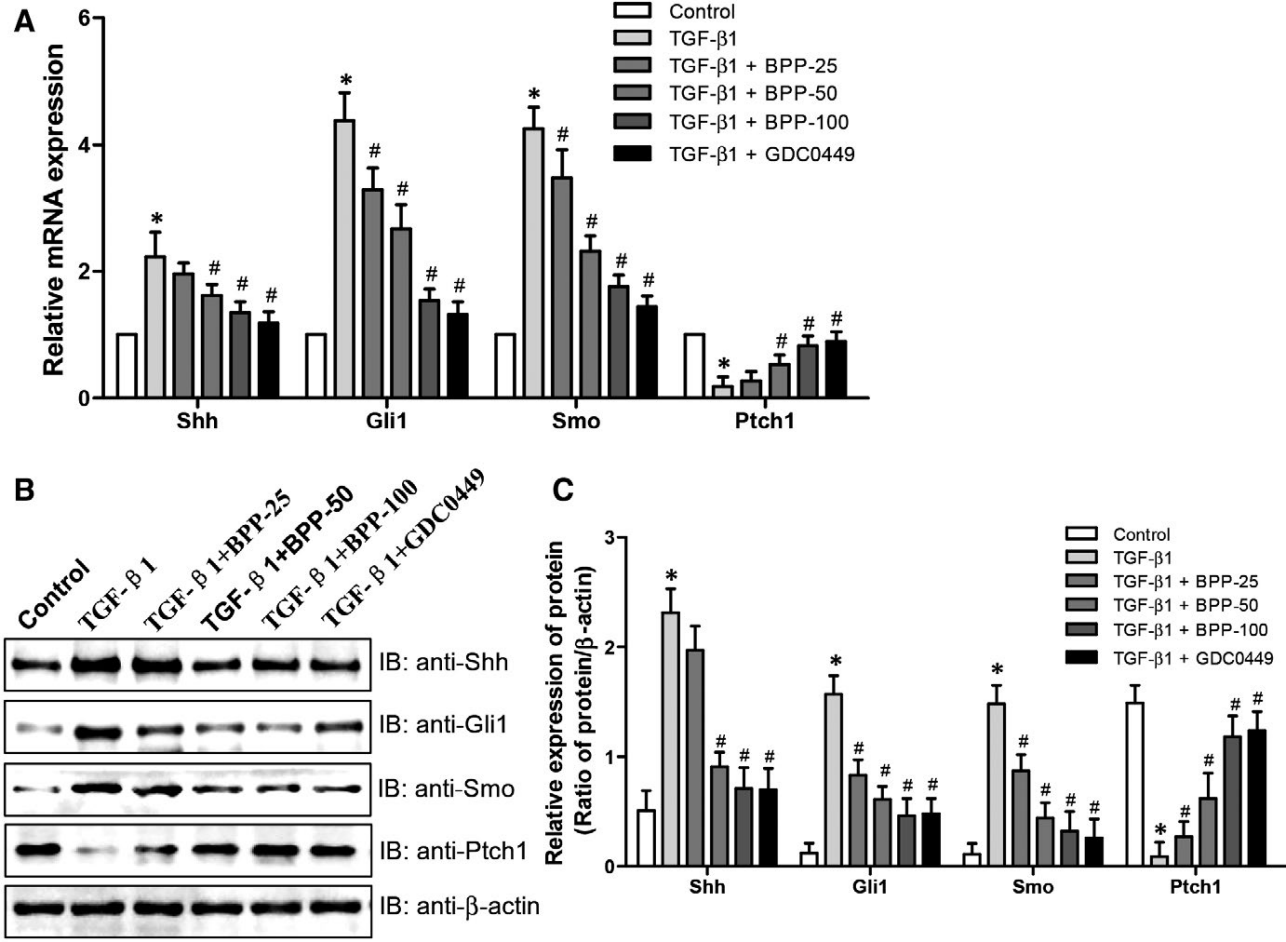
Effects of BPP on the SHH signalling pathway in TGF‐β1‐treated HK‐2 cells. (A) The mRNA expression levels of Shh, Gli1, Ptch1 and Smo were determined by quantitative real‐time PCR. (B) The representative protein expression levels of Shh, Gli1, Ptch1 and Smo were examined by western blot. Antibody against β‐actin was used as an internal control. (C) The relative protein expression levels of Shh, Gli1, Ptch1 and Smo were analysed based on western blot results. All the experiments were performed in triplicate. Data in (A) and (C) were expressed as mean ± SD, *P < .05 compared with the control group, ^#^ *P* < .05 compared with the TGF‐β1‐treated group, n = 3. BPP‐25, 25 μg/mL of BPP; BPP‐50, 50 μg/mL of BPP; BPP‐100, 100 μg/mL of BPP. GDC0449 was used as positive control

Our in vivo studies suggested that the SHH signalling pathway may lie a crucial position in the signalling cascade involved in mediation of anti‐fibrotic effects of BPP. For confirming this hypothesis, we examined the mRNA and protein levels of Shh, Gli1, Smo and Ptch1 in TGF‐β1‐treated HK‐2 cells. As shown in Figure 7, the mRNA (Figure 7A) and protein (Figure 7B) expression levels of Shh, Gli1 and Smo were significantly enhanced, whereas the mRNA and protein levels of Ptch1 were simultaneously reduced in TGF‐β1‐treated HK‐2 cells. However, BPP treatment observably and significantly attenuated the levels of Shh, Gli1, Smo and up‐regulated mRNA and protein levels of Ptch1 expression, respectively. These results are consistent with those of the effects of BPPs on the levels of these genes in renal tissues of UUO mice. Thus, both the in vivo and in vitro experimental results confirm each other and clearly indicate that the inhibition of SHH signalling pathway may be one of the key mechanisms accounting for the anti‐fibrotic effect of BPPs in the kidney.

## 4 DISCUSSIONS

RIF is one of the main causes for the renal failure. However, effective medicines for treating RIF are currently unavailable and needed. Thus, in this study, to confirm the renal anti‐fibrosis effect of BPP, we investigated the effects of BPPs on the renal tubulointerstitial injury score, fibroblast activation, collagen expression and ECM accumulation in UUO mice. Our results clearly showed that BPPs could ameliorate renal interstitial damage dose‐dependently. Especially, the interstitial injury score of the mice in BPP‐H (450 mg/kg.d) group was significantly decreased as compared with that of UUO group and was nearly close to that of the positive control group. To gain significant insights into the cellular and molecular mechanisms underlying the anti‐fibrotic effects of BPPs, we investigated the effects of BPPs on the mRNA and protein expression levels of genes involved in ECM deposition and degradation, EMT and SHH signalling pathway in renal tissue of UUO mice. We also confirmed and extended the in vivo results of the effects of BPP on genes involved in ECM accumulation, EMT and SHH signalling pathway in TGF‐β1‐treated HK‐2 cells. These experiments led us to make several interesting and important findings.

Firstly, we found that BPPs significantly reduced the accumulation and deposition of tubulointerstitial ECM components, including collagens I, III and IV, and the hallmarks of fibroblast activation, fibronectin (FN) and α‐SMA. It is well known that the accumulation and deposition of ECM is the fundament of renal fibrogenesis.^22^ The Masson's trichrome staining results showed that BPP administration remarkably reduced the Masson's staining positive region and accumulation of tubulointerstitial ECM (Figure 1 A and B). Collagens I, III and IV are the important ECM constituents, which are deposited in the kidneys during tubulointerstitial fibrogenesis. Our results obtained from both RT‐PCR and Western blot indicated that BPP treatment significantly reduced their mRNA and protein expression levels in the kidneys of UUO mice (Figure 2 A‐E). The renal interstitial fibroblasts are regarded as the essential source of fibrillar matrix, including collagens I, III and IV. Moreover, FN and α‐SMA are the hallmarks of fibroblast activation. Our results from immunohistochemistry and quantitative RT‐PCR displayed that BPP administration substantially decreased the expression levels of both FN and α‐SMA in the obstructed kidney (Figure 2 F‐H). Additionally, above‐mentioned results could also be observed in TGF‐β1‐treated HK‐2 cells in vitro (Figure 6).

Secondly, we found that BBP treatment simultaneously up‐regulated the expression levels of MMP2 and MMP9 involved in alleviating ECM and down‐regulated TIMP1 and TIMP2 involved in inhibition of matrix metalloproteinases (MMPs) in both the renal tissues of UUO mice and TGF‐β1‐treated HK‐2 cells. It was previously reported that ECM alleviation was mediated mainly by MMPs, including matrilysins and collagenases, etc,^23^, ^24^ especially MMP2 and MMP9, which are closely correlated with renal fibrosis.^25^ However, the activities of MMPs are regulated chiefly by TIMPs. In the family of TIMPs, both TIMP1 and TIMP2 are able to inhibit the activities of MMPs, thus playing a crucial role to maintain the balance between accumulation and degradation of ECM.^26^ For instance, Sharma et al had reported that collagen IV degradation was suppressed when TIMPs were up‐regulated whereas MMPs were down‐regulated in renal fibrosis, thus promoting ECM accumulation.^27^ Our current results showed that in the kidneys of UUO mice, the mRNA and protein expression levels of MMP2 and MMP9 were declined, whereas those of TIMP1 and TIMP2 were increased, indicating that the balance between accumulation and degradation of ECM is destroyed and towards the accumulation of ECM in renal tissue of UUO mice. This is consistent with the Masson's trichrome staining results showing the obvious collagen deposition in the obstructed kidneys. After being treated with BPPs, the mRNA and protein expression levels of MMP2 and MMP9 were elevated, whereas those of TIMP1 and TIMP2 were reduced (Figure 3). Moreover, similar results were also observed in TGF‐β1‐treated HK‐2 cells in vitro (Figure 6). Altogether, these results suggest that during the progression of fibrogenesis in UUO mice and in TGF‐β1‐treated HK‐2 cells, ECM deposition was usually associated with changes in expression levels of MMPs and TIMPs. BPPs could ameliorate the renal fibrosis through regulating the balance between TIMPs and MMPs. BPP treatment reduced the disposition and accumulation of ECM by enhancing ECM degradation via up‐regulating the expression levels of MMPs and down‐regulating the expression levels of TIMPs, resulting in the reversion of the balance between degradation and disposition of ECM towards degradation, and thus, improving renal fibrosis.

Thirdly, we found that BBP treatment could alleviate EMT in the kidneys of UUO mice via the depression of transcriptional factors involved in EMT. The EMT occurring in the renal tubular epithelia is an important process during the renal fibrogenesis and manifested by the loss of epithelial phenotype and acquirement of mesenchymal phenotype, characterized by the declined expression of E‐cadherin and the enhanced expression of N‐cadherin. The decreased E‐cadherin level can lead to the decreased cell adhesion, enabling cells to acquire the characteristics of easy invasion and metastasis. Loss of E‐cadherin expression has been considered as the most significant characteristic of EMT. At the same time, cells acquired stromal phenotypes, such as the increased expression of vimentin and N‐cadherin. Expression of N‐cadherin in epithelial cells induces changes in morphology to a fibroblastic phenotype, rendering the cells more motile and invasive. Growing lines of evidence have demonstrated that EMT is the key pathway for the origin of renal interstitial myofibroblasts, suggesting that EMT can be the potential target for inhibiting and even reversing the progression of renal fibrosis.^8^, ^9^ In our current study, we investigated the effects of BPP on the mRNA and protein levels of epithelial marker E‐cadherin, and the mesenchymal markers, N‐cadherin and vimentin in the kidneys of UUO mice. We observed that mRNA and protein levels of E‐cadherin were significantly lower whereas those of N‐cadherin and vimentin were significantly higher in UUO mice as compared to those of the mice in Sham group. After being treated with BPP, the mRNA and protein levels of E‐cadherin were substantially up‐regulated whereas those of N‐Cadherin and vimentin were significantly down‐regulated in both the kidneys of UUO mice and TGF‐β1‐treated HK‐2 cells (Figure 4 and Figure 6), indicating that BPP administration can inhibit the progression of renal EMT. More notably, we also observed that mRNA and protein levels of the EMT‐related transcriptional factors, including Snail, Twist and ZEB1, were significantly higher in kidneys of UUO mice. BPP treatment could effectively reduce the mRNA and protein levels of these EMT‐related transcriptional factors in the kidney of UUO mice or in TGF‐β1‐treated HK‐2 cells. All of these results indicate that the protective effects of BPP on the renal fibrosis and EMT in UUO mice or TGF‐β1‐induced HK‐2 cells are at least partially achieved by inhibiting the expression levels of transcription factors, including Snail, Twist and ZEB1.

Fourthly, we found that BBP treatment could dose‐dependently and significantly suppressed the levels of Shh, Gli1 and Smo, the important factors involved in the SHH signalling pathway, and enhanced the levels of Ptch1, the inhibitor of Shh signalling pathway. More and more studies have reported that Shh signalling pathway plays a crucial role in many diseases.^28^, ^29^ The functions of the Shh signalling pathway in the occurrence and development of CKD have also been studied extensively. Recent research has already shown that abnormal activation of the Shh signalling pathway could lead to renal fibrogenesis.^30^ Additionally, several other groups have also confirmed that the Shh signalling pathway is abnormally activated during the EMT process.^31^, ^32^ Moreover, the Shh signalling pathway contributes to the activation of fibroblasts and the progression of EMT in kidney through regulating the expression levels of several fibrosis‐related genes, thus resulting in the ECM accumulation and the fibrogenesis. When the secreted Shh ligand reaches its target cell, it binds to the cell surface receptor Ptch1 with a high affinity, thus initiating the signal. In the absence of Shh, Ptch1 generally inhibits Smo activity. On the contrary, the inhibition of Smo can be alleviated when Shh is combined with Ptch1.^33^ The accumulation of Smo receptors opened a complex network, activated Gli1 and induced the expression of α‐SMA, desmin, Snail1, fibronectin and collagen I, which promoted the proliferation of renal fibroblasts and stimulated the induction of a variety of proliferation related genes.^12^, ^34^

The current study provided further the experimental evidence showing that Shh signalling pathway is activated during the pathogenesis of the renal fibrosis. Our results displayed that the mRNA and protein levels of Shh, Gli1 and Smo, which are important factors involved in Shh signalling pathway, were significantly elevated whereas those of Ptch1, an inhibitor of Shh signalling, were significantly lower in kidneys of UUO mice. BPP treatment notably and significantly declined the mRNA and protein levels of Shh, Gli1 and Smo and enhanced the expression of Ptch1 in the kidney of UUO mice or in TGF‐β1‐treated HK‐2 cells (Figure 5 and Figure 7). Thus, the anti‐fibrotic effect of BPP in the mouse kidney may be closely related to the inhibition of the Shh signalling pathway by down‐regulating the levels of important activating transcriptional factors and up‐regulating the levels of negative regulators, such as Ptch1, involved in this pathway. Of course, further study is needed to elucidate the detailed cellular and molecular mechanisms. Considering the crucial role of the Shh signalling pathway in renal fibrosis, taking this pathway as a potential target might be a prospective breakthrough and effective way for the inhibition and reversion of the fibrosis of CKD.

*Balanophora polyandra Griff* is a natural medicinal mushroom and has been known to have numerous medicinal values. More recently, our group has demonstrated that BPPs significantly inhibited ovarian cancer cell proliferation via a P53‐mediated pathway.^19^ The present study clearly demonstrated that BPPs could also significantly ameliorate kidney fibrosis and EMT via inhibiting the Shh signalling pathway. These studies indicate the nutritional and medicinal values and potential applications of *Balanophora polyandra Griff* is as a functional food in clinical treatment for patients with chronic kidney disease and cancer. This prospective is worthy for further exploration.

The doses of *Balanophora polyandra Griff* crude drug reported in Chinese literature are in the range of 9‐15 grams. We used the maximum human dose (15g/70kg) to calculate the conversion ratio of the body surface area between the human body (70kg) and the mouse (20g), which is 387.9, that is, the equivalent dose (mg/g) of the mouse is 38.67mg/20g. In addition, we calculated that the extraction rate of total extract of Balanophora polyandra Griff polysaccharides (BPP), which was 0.528. Therefore, the maximum dose of BPP in mice was calculated by the drug extraction rate, which was 1019.04 mg/kg. Therefore, we determined that the highest dose of BPP was 1000 mg/kg while the lowest dose was 1/4 of the high dose, which was 250mg/kg. Finally, according to the experimental results of animal tolerance, the dose of BPP was adjusted to: BPP‐L, low dose of BPP (150mg/kg.d); BPP‐H, high dose of BPP(450mg/kg.d).

## 5 CONCLUSIONS

In conclusion, these in vitro and in vivo experiments preliminarily demonstrated that BPP treatment could inhibit the hedgehog signalling pathway and EMT induction, resulting in the reduced ECM accumulation in the kidneys. These results also implicate that BPPs could be used as a potential therapeutic agent for preventing kidney fibrosis. Obviously, its potential applications in clinical treatment of chronic kidney disease still need further exploration.

## CONFLICT OF INTEREST

There is no declaration of any conflict of interest.

## AUTHOR CONTRIBUTIONS

**Luoying Li:** Writing‐original draft (lead); Writing‐review & editing (equal). **Gang Zhou:** Project administration (lead); Resources (lead). **Rui Fu:** Investigation (equal); Methodology (equal). **Yumin He:** Methodology (lead); Resources (equal). **Li Xiao:** Conceptualization (equal); Project administration (equal). **Fan Peng:** Data curation (equal); Investigation (equal). **Chengfu Yuan:** Conceptualization (lead); Funding acquisition (lead); Resources (lead); Writing‐original draft (lead); Writing‐review & editing (lead).

## Supporting information

Table S1
