# Supplementary material for: Polysaccharides extracted from balanophora polyandra Griff (BPP) ameliorate renal Fibrosis and EMT via inhibiting the Hedgehog pathway
Source: J Cell Mol Med. 2021 Jan 28;25(6):2828–40. doi: 10.1111/jcmm.16313 (PMC7957266; doi:10.1111/jcmm.16313)
Supplement: Supplementary file 1 — Table S1 [file JCMM-25-2828-s001.docx]

**Supplementary table The primer sequences used for real-time PCR**

| Gene | Forward primer | Reverse primer |
| --- | --- | --- |
| E-cadherin(mouse) | cggccaaggagctgacaa | atgggatcctccaccgct |
| N-cadherin(mouse) | agctgctgacaacgaccc | ttcagtcgtcaccaccgc |
| Vimentin(mouse) | gctgcaggcccagattca | accattcctcggcctcct |
| Snail(mouse) | ctggtaccccaagtgcgg | gccgaggtggacgagaag |
| Twist(mouse) | ccatgtccggacccacac | gccggactcttggtgctt |
| ZEB1(mouse) | ttcagctgctccctgtgc | cttgaaggccttcccgca |
| TIMP1(mouse) | gtggggtgtgcacagtgt | aagcctggattccgtggc |
| TIMP2(mouse) | gccccctcttcagcagtg | cgtgtcccagggcacaat |
| MMP2(mouse) | gacaagcccacaggtccc | ctggtcagtggcttgggg |
| MMP9(mouse) | gtggtgtcccagacgtgg | tcgatcatgtctcgcggc |
| Fibronectin(mouse) | cagcgaaggcctgaacca | agccagcgtcagacaacc |
| Collagen I(mouse) | gctcctcttaggggccact | ccacgtctcaccattgggg |
| Collagen III(mouse) | gaggaaagggccctgctg | aactccgtcagcacctgc |
| Collagen IV(mouse) | gccaggtgacacaggacc | cacctcgtcctcctggga |
| α-SMA(mouse) | caggctgtgctgtccctc | cagccaagtccagacgca |
| Shh(mouse) | tctgcaacggaagcgagg | gctggacttgaccgccat |
| Gli1(mouse) | ccaagccaactttatgtcaggg | agcccgcttctttgttaatttga |
| Smo(mouse) | cgctcttcacactggcca | catgaactgggccagcca |
| Ptch1(mouse) | aaagaactgcggcaagtttttg | cttctcctatcttctgacgggt |
| GAPDH(mouse) | aactttggcattgtggaagg | acacattgggggtaggaaca |
| Collagen I(human) | gctcctggcagcaaagga | cagtgggtccgggttcac |
| α-SMA(human) | gagcttcgtgttgcccct | agttgtgcgtccagaggc |
| MMP9(human) | gctgtgcgtcttcccctt | gccccacttcttgtcgct |
| TIMP1(human) | cacagacggccttctgca | cagcggcatcccctaagg |
| E-cadherin(human) | gtcaccactgggctggac | aatccaagcccgtggtgg |
| N-cadherin(human) | ctgcagcagcctgacact | gggtcattgtcagccgct |
| Vimentin(human) | ccgcaccaacgagaaggt | ttggcccttgagctgctc |
| Snail(human) | ccatgtccggacccacac | gccggactcttggtgctt |
| Twist(human) | agcctgagcaacagcgag | agacttcttgccgcgctt |
| ZEB1(human) | caccaagtgccaacccca | ctgcagggctgaccgtag |
| Shh(human) | ctcgtctcctcgctgctg | tggcgcctagggtcttct |
| Gli1(human) | acctgaagacgcacctgc | tgcttggctcggtcactg |
| Smo(human) | cagttcaggccagtgcga | cagggtgaagagcgtgca |
| Ptch1(human) | agccgcgataagcccatc | tcggctttgtcgtggacc |
| GAPDH(human) | gagtcaacggatttggtcgt | ttgattttggagggatctcg |
